# Psychometric testing of the breastfeeding self-efficacy scale to measure exclusive breastfeeding in African American women: a cross-sectional study

**DOI:** 10.3389/fpubh.2023.1196510

**Published:** 2023-09-25

**Authors:** Tumilara Aderibigbe, Stephen Walsh, Wendy A. Henderson, Ruth F. Lucas

**Affiliations:** ^1^School of Nursing, University of Connecticut, Storrs, CT, United States; ^2^College of Nursing, University of Utah, Salt Lake City, UT, United States; ^3^School of Medicine, University of Connecticut, Storrs, CT, United States

**Keywords:** African American, cross-sectional, exclusive breastfeeding, self-efficacy, validation, women, prenatal breastfeeding self-efficacy

## Abstract

**Background:**

In United States, African American women are the least likely group to breastfeed exclusively compared with Hispanic and non-Hispanic White women. It is crucial to examine the perceived confidence of African American women towards practicing exclusive breastfeeding. Previous studies have examined breastfeeding self-efficacy and other factors influencing exclusive breastfeeding. However, there is no research on exclusive breastfeeding self-efficacy of this population. The purpose of this study was to examine the validity and reliability of the breastfeeding self-efficacy scale to measure exclusive breastfeeding, and the relationship between exclusive breastfeeding self-efficacy and general self-efficacy and demographic variables in African American women.

**Methods:**

Descriptive cross-sectional design was used. A convenience sample of 53 pregnant African American women completed an online survey. Construct and criterion-related validity were assessed and reliability of the breastfeeding self-efficacy scale to measure exclusive breastfeeding (BSES-EBF) was examined using Cronbach’s reliability. The general self-efficacy scale measured general self-efficacy. Descriptive statistics, bivariate correlation and non-parametric analyses were performed using statistical package for social sciences (v.28).

**Results:**

The breastfeeding self-efficacy to measure exclusive breastfeeding scale had a Cronbach’s alpha score of 0.907. One principal component was extracted from the BSES-EBF scale, with an Eigenvalue of 5.271 and which explained 58.57% of the variance in the instrument. The mean prenatal exclusive breastfeeding self-efficacy of participants was 35.15 (±7.41) from a range of 9 to 45. Exclusive breastfeeding was significantly associated with general self-efficacy (*r* = 0.503, *p* ≤ 0.001) and exclusive breastfeeding intention (*p* = 0.034).

**Conclusion:**

Breastfeeding self-efficacy scale to measure exclusive breastfeeding is a valid and reliable tool to measure exclusive breastfeeding self-efficacy in African American women. African American women had high exclusive breastfeeding self-efficacy (internal motivation). Hence, there is a need to address breastfeeding barriers and provide access to culturally sensitive support (external motivation) to increase exclusive breastfeeding in African American women.

## 1. Introduction

Infant nutrition in the first 1,000 days is important, as it is a crucial period of development (1). Failure to provide adequate nutrition during this period may result in adverse health outcomes including diarrhoea, pneumonia, decreased vaccine efficacy (2), and stunting, leading to poor cognitive performance (3). To this end, the World Health Organization recommended exclusive breastfeeding (EBF) for 6 months of life to promote the health of infants (4). In many countries, women who followed this recommendation reported health benefits not just for their infants but for them as well; these include adequate weight gain and absence of hospitalization for infants, weight maintenance, prevention of conception and hormonal imbalance, cardiac disease and cancer, for women (5, 6).

Racial disparities exist in EBF rates in the United States. African American (AA) women are the least likely group to intend (57%), initiate (61%) and maintain breastfeeding (6.4 weeks) compared with Spanish-speaking Hispanic (92, 91%, 17.1 weeks) and non-Hispanic White (77, 78%, 16.5 weeks) women, respectively, Mckinney et al. (7). Indeed, among women who gave birth in 2019, only 19.1% of AA women breastfed exclusively for 6 months compared with 23.5 and 26.9% of Hispanic and non-Hispanic White women, respectively, Centers for Disease Control and Prevention (8). This low rate of EBF among AA women may be attributed to maternal (attitude, breastfeeding self-efficacy) and contextual (socioeconomic status, generational trauma of wet nursing) factors (9, 10).

Breastfeeding self-efficacy refers to a woman’s confidence in her ability to breastfeed her infant (11). The role of breastfeeding self-efficacy towards achieving and sustaining both breastfeeding (12, 13) and EBF (14–17) has been established in many studies. While the available instruments for measuring breastfeeding self-efficacy measure all or part of the breastfeeding self-efficacy construct (18, 19), these instruments may not be the most appropriate to predict EBF, especially because Bandura had argued that self-efficacy should be examined using a behaviour-specific approach (20). As such, an instrument that specifically measures EBF self-efficacy may more precisely measure the relationship between EBF self-efficacy and EBF. Prenatal breastfeeding intention has been identified as a strong predictor of breastfeeding and EBF because it reflects maternal sociodemographic characteristics, maternal knowledge, and attitude towards breastfeeding and social norms (21, 22). In addition, learning about breastfeeding (knowledge) may promote a woman’s breastfeeding intention, which can turn into a behaviour later (practice) (23). There is a strong relationship between prenatal breastfeeding intention and prenatal breastfeeding self-efficacy. Both variables were found to mediate breastfeeding and EBF duration in first and second child (24). In addition, the prenatal rating of efficacy in preparation to breastfeed scale was highly correlated with breastfeeding intention (19). Similarly, women who planned to breastfeed had higher prenatal breastfeeding self-efficacy scores compared to women who planned to formula feed their infants (25).

Given the low rate of EBF in AA women, it is important to examine EBF self-efficacy and identify its predictors in this population. Several studies reported that AA women have lower prenatal breastfeeding self-efficacy compared to non-Hispanic White women (26). One study also reported that AA women had low postnatal breastfeeding self-efficacy (27). Boateng and colleagues developed a new tool, the breastfeeding self-efficacy scale to measure exclusive breastfeeding (BSES-EBF) (28). The tool, originally validated using a longitudinal design among women in Uganda (28), was adapted and validated using a cross-sectional design among women in Egypt (29). Women in Uganda had a mean BSES-EBF score of 30.65 whereas, about 50.2% of women had high BSES-EBF scores in Egypt (29). For African women, breastfeeding is considered a norm (30). On the other hand, early supplementation is common among AA women because of the generational trauma of wet nursing (9, 31), and the belief that formula is more quality than breast milk (32). This may be a plausible reason for the lower breastfeeding self-efficacy among AA women compared to African women (27). Boateng and colleagues recommended that future studies should test their BSES-EBF tool in a new population (with low EBF rates) and examine the construct validity of the instrument by assessing the true correlation between the BSES-EBF scale scores and related constructs. No study has validated this tool among AA women in the United States; hence this study will fill the research gap. The aim of this study was to assess psychometric properties of the BSES-EBF tool, and the relationship between EBF self-efficacy and general self-efficacy, and demographic characteristics among pregnant AA women. The research questions are: (1) what is the relationship between exclusive breastfeeding intention and exclusive breastfeeding self-efficacy? (2) what is the relationship between parity and exclusive breastfeeding self-efficacy? and (3) what is the relationship between general self-efficacy and exclusive breastfeeding self-efficacy?

Dennis’ breastfeeding self-efficacy theory, one of the two most used theories that supported interventions to promote EBF, guided this study (11, 33). The breastfeeding self-efficacy theory originated from Bandura’s social cognitive theory (34). Self-efficacy, according to Bandura, is the belief in one’s ability to organize and accomplish tasks required to manage prospective situations (20). Self-efficacy comprises outcome expectancy (the perception that a behaviour will produce a specific outcome) and self-efficacy expectancy (belief that one can perform a behaviour that will result in a desired outcome) (20). Thus, to be identified as having self-efficacy, a person must believe that performing a behaviour will result in a desired outcome and be confident in one’s ability to perform the behaviour. Dennis proposed that breastfeeding self-efficacy plays an important role in breastfeeding duration and emphasized that it also predicts (a) a woman’s decision to breastfeed, (b) the intensity of effort she will expend, (c) probability that she will persevere in her efforts until mastery is achieved, (d) whether she will have self-enhancing or self-defeating thought patterns, and (e) how she will respond emotionally to difficulties (11, 35). In AA women, it is necessary to examine predictors of EBF self-efficacy, considering their low socioeconomic status, generational trauma associated with wet nursing, and other challenges they may face while attempting to breastfeed exclusively.

## 2. Methods

### 2.1. Design and setting

Descriptive cross-sectional design was used in the study to collect data from July 8, 2021, to February 13, 2022 (36). Research setting was the United States, and data were collected online due to Covid-19 pandemic. Research advertisement was posted on RL’s website and social media platforms including Facebook, LinkedIn, Instagram, and Twitter. Most of the participants (90%) were recruited from Facebook using ads targeted at the research population.

### 2.2. Sample

The target population for the study were AA women living in the United States. AA women population were chosen because they have lower EBF rates compared with other minority ethnic groups in United States (37). Inclusion criteria were as follows: (a) English language comprehension: questionnaires were written in English, (b) access to internet: survey was delivered online through UConn Qualtrics, (c) currently pregnant: prenatal exclusive breastfeeding self-efficacy is the outcome variable, and (d) age range 18–50 years: eligible age for provision of informed consent for participation in research in United States is 18 years (38). Sample size for the study was calculated using G*power software (39). The correlation coefficient method used for sample size calculation in a previous validation study was adopted in this study (40). Hence, the correlation between exclusive breastfeeding self-efficacy, measured using BSES-EBF, and exclusive breastfeeding social support (range 0.23–0.47) served as a reference for the sample size calculation in the present study (28). The midpoint of the correlation range is 0.35, hence, to detect a correlation of 0.35 from a two-tailed test, power (1−β) of 0.8 and alpha of 0.05 yielded a sample size of 59.

### 2.3. Measurements

Independent variables were general self-efficacy and demographic characteristics while EBF self-efficacy was the dependent variable.

#### 2.3.1. Demographic characteristics and infant feeding method

Demographic information in the survey include age, marital status, parity, highest level of education, employment, and intention to breastfeed exclusively.

#### 2.3.2. Exclusive breastfeeding self-efficacy

Exclusive breastfeeding self-efficacy, defined as a woman’s confidence in her ability to breastfeed exclusively was the main outcome variable in this study. The breastfeeding self-efficacy scale to measure exclusive breastfeeding (BSES-EBF) (28), which originated from the short form of Dennis’ breastfeeding self-efficacy scale (35), was used to measure EBF self-efficacy. BSES-EBF is valid and reliable, with Cronbach’s alpha coefficients of 0.82 and 0.85, and 0.77 and 0.79 at 3 months for the Cognitive and Functional sub-scales of the BSES-EBF, respectively, Boateng et al. (28). The instrument contains 9 items on a 5-point Likert scale ranging from 1 (not at all confident) to 5 (very confident) with higher scores indicating greater confidence to practice exclusive breastfeeding. The minimum and maximum scale scores are 9 and 45, respectively. BSES-EBF was positively correlated with exclusive breastfeeding social support (*r* = 0.28, *p* = 0.001) and negatively correlated with depression (*r* = −0.14, *p* = 0.05) (28). In the present study, EBF self-efficacy scores were grouped into three categories: low (0–15), medium (16–30), and high (31–45) scores for descriptive analysis.

We reviewed items on the BSES-EBF to appraise the tool’s appropriateness for AA women since the tool was developed in Uganda. As recommended by Boateng and colleagues, the BSES-EBF is suitable for population with low EBF rates (28). AA women have lower rate of exclusive breastfeeding compared to any type of breastfeeding (8). In addition, items on the instrument were written in a simple language that is easily comprehensible for people with formal education. More than half of AA women have at least high school education (41). Therefore, we determined that items on the BSES-EBF are culturally appropriate for AA women.

#### 2.3.3. General self-efficacy

General self-efficacy is the belief in one’s ability to cope with stressful situations (42). This variable was measured using the General self-efficacy scale developed by Schwarzer and Jerusalem (43). The general self-efficacy scale is valid and reliable, positively correlated with optimism, negatively correlated with stress and depression, with Cronbach’s alpha scores between 0.76–0.9 (43). The instrument contains 10 items on a 4-point Likert scale ranging from 1 (not at all true) to 4 (very true). The minimum and maximum scale scores are 10 and 40, respectively. In the present study, general self-efficacy scores were grouped into two categories: low (0–20) and high (21–40) for descriptive analysis.

### 2.4. Statistical analysis

Data were analyzed using Statistical Package for Social Sciences (v.28). Seventy-six women responded to the survey however, only 55 women met eligibility criteria, and two women did not provide any response to the questionnaire. One of these two women did not provide a response to the question about provision of informed consent for the study and the other, who provided consent did not answer any question in the survey. Therefore, 53 participants provided data for the study nonetheless, one of the 53 participants had missing responses to five items in the general self-efficacy instrument. Hence data analysis involving general self-efficacy was conducted with 52 complete responses. EBF self-efficacy and general self-efficacy were not normally distributed in our sample (skewed to the left). The Shapiro–Wilk test further revealed significant *p*-values for both variables, affirming their skewness: *p* = 0.03 and *p* = 0.014 for EBF self-efficacy and general self-efficacy, respectively. Therefore, the relationship between EBF self-efficacy and general self-efficacy was assessed using correlation analysis (Spearman’s correlation) while the relationships between EBF self-efficacy and other demographic characteristics were assessed using Kruskal-Wallis’ test. Both tests use rank of rather than value of observations in the analyses. Spearman’s rank-order correlation is the preferred test when Pearson’s correlation test is unsuitable due to non-normality of data (44). Similarly, Kruskal-Wallis’ test is the preferred test when one-way ANOVA is unsuitable due to non-normality of data (45). For descriptive statistics, means [standard deviations (SD)] and frequencies (percentages) of variables were computed.

### 2.5. Ethics approval

The study was approved by the Institutional Review Board at University of Connecticut in May 2021 (approval number: X21-0090). The survey included the information sheet which also contained a question on informed consent. Only participants who provided informed consent were granted access to the survey.

## 3. Results

### 3.1. Participant characteristics

The majority of participants were within the age group 18–30 years, had given birth to one or two children (60.4%), and planned to breastfeed exclusively after birth (81.1%) (Table 1). Only 28.4% of participants had a college degree. Early in the study, a comment posted on the Facebook ad warned women not to participate in our study and making reference to the Tuskegee study which may have limited responses to the study.

**Table 1:** Descriptive statistics (*n* = 53).

Characteristics	Frequency (*N*)	Percentage (%)
Age (in years)		
18–30	27	50.9
31–40	25	47.2
41–50	1	1.9
Marital status		
Single	27	50.9
Married	16	30.2
Separated	3	5.7
Divorced	4	7.5
Prefer not to answer	3	5.7
Parity		
0	5	9.4
1–2	32	60.4
3–4	13	24.5
5 or more	3	5.7
Education		
Grades 1–11	4	7.5
High school			1	1.9
High school diploma or GED	10	18.9
Some college	17	32.1
Graduated 2-year college	6	11.3
Graduated 4-year college	10	18.9
Masters	3	5.7
PhD	2	3.8
Employment		
Full-time	18	34.0
Part-time	17	32.1
Unemployed	14	26.4
Student	4	7.5
Exclusive breastfeeding intention		
Formula feed only	2	3.8
Breastfeed only	43	81.1
Formula feed and breastfeed	7	13.2
Undecided	1	1.9
General self-efficacy, mean ± SD	33.56 (4.67)	
Exclusive breastfeeding self-efficacy, mean ± SD	35.15 (7.41)	

### 3.2. Construct (factorial) and criterion-related validity

**Figure 1:**
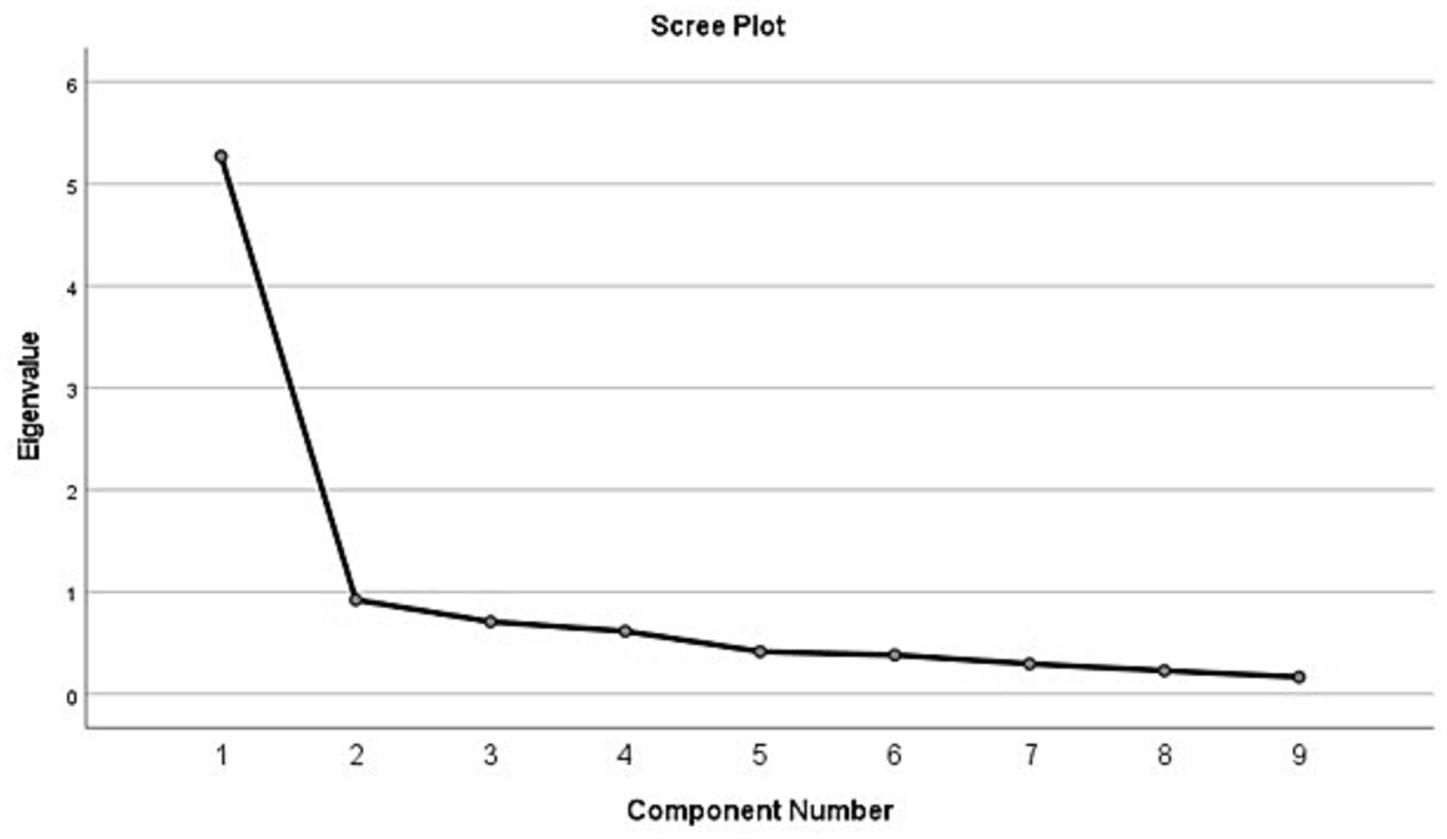
Scree plot of the 9-item BSES-EBF scale.

Principal factor analysis was conducted to identify latent variable(s) underlying the BSES-EBF scale. Results from the principal component extraction showed that the instrument had only one component that met Kaiser’s criterion (Eigenvalue>1) (46, 47). The principal component had an Eigenvalue of 5.271 and explained 58.57% of the variance (Figure 1). All the nine items in the BSES-EBF instrument loaded strongly and positively on the principal component (range: 0.571–0.898) (Table 2). All factor loadings were greater than 0.4, suggesting that all items in the instrument are stable, as such, it was not necessary to repeat reliability analysis (which is required only in cases where items with loadings <0.4 were removed) (48). EBF self-efficacy was significantly associated with general self-efficacy and intention to breastfeed exclusively in this study, implying that the instrument has construct and criterion-related validity, respectively.

**Table 2:** BSES-EBF items and their principal component factor loadings.

Items	Loadings
I will always know whether my baby is getting enough milk.	0.787
I will always be able to give my baby breast milk without using animal milk, formula, or other liquids or foods as a supplement.	0.771
I will be able to continue exclusive breastfeeding for as long as I want.	0.742
I will always be satisfied with my exclusive breastfeeding experience.	0.757
I will always be able to deal with the fact that breastfeeding can be time consuming.	0.724
I will continue to breastfeed my baby for every feeding.	0.841
I will always be able to keep up with my baby’s breastfeeding demands.	0.898
I will always exclusively breastfeed without my baby receiving even a drop of water or any liquid.	0.755
I will always stop someone from trying to feed my baby liquids or foods other than breast milk, including purchased baby foods (e.g., infant formula, milk, porridge, juice, and tea [whatever is given]), before 6 months of age.	0.571

### 3.3. Internal consistency reliability

In the present study, Cronbach’s alpha coefficient was used to assess reliability of instruments. BSES-EBF scale and general self-efficacy scale had Cronbach’s alpha scores of 0.907 and 0.888, respectively. Cronbach’s alpha of 0.7 and above is generally considered acceptable (49, 50). BSES-EBF and general self-efficacy scales are reliable to measure EBF self-efficacy and general self-efficacy in AA women. Because the BSES-EBF items were relatively small, we examined items as a whole, not the sub-scales as in Boateng et al. (28).

### 3.4. Predictors of exclusive breastfeeding self-efficacy

**Figure 2:**
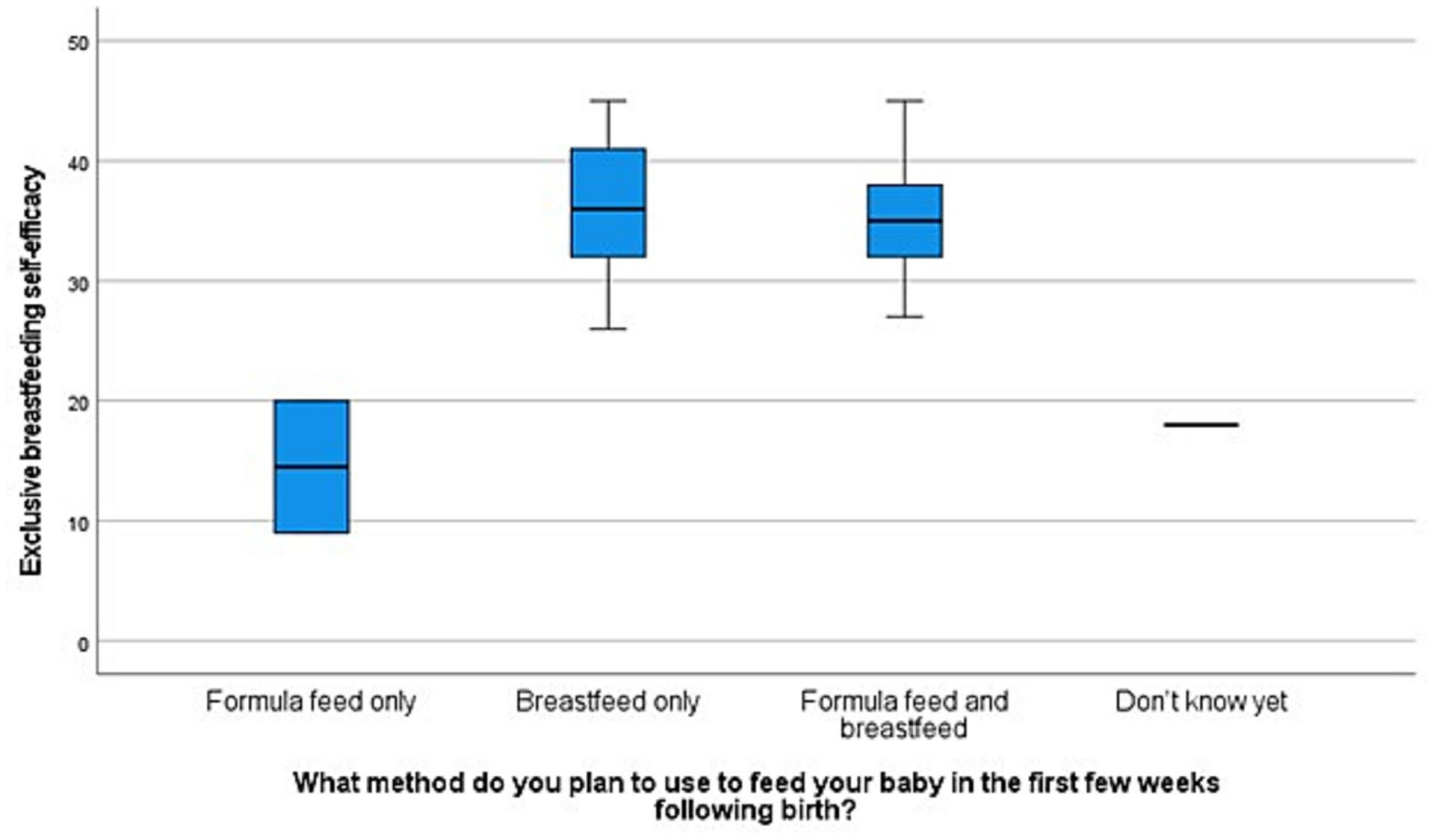
Box plot of exclusive breastfeeding self-efficacy scores according to planned feeding method.

**Figure 3:**
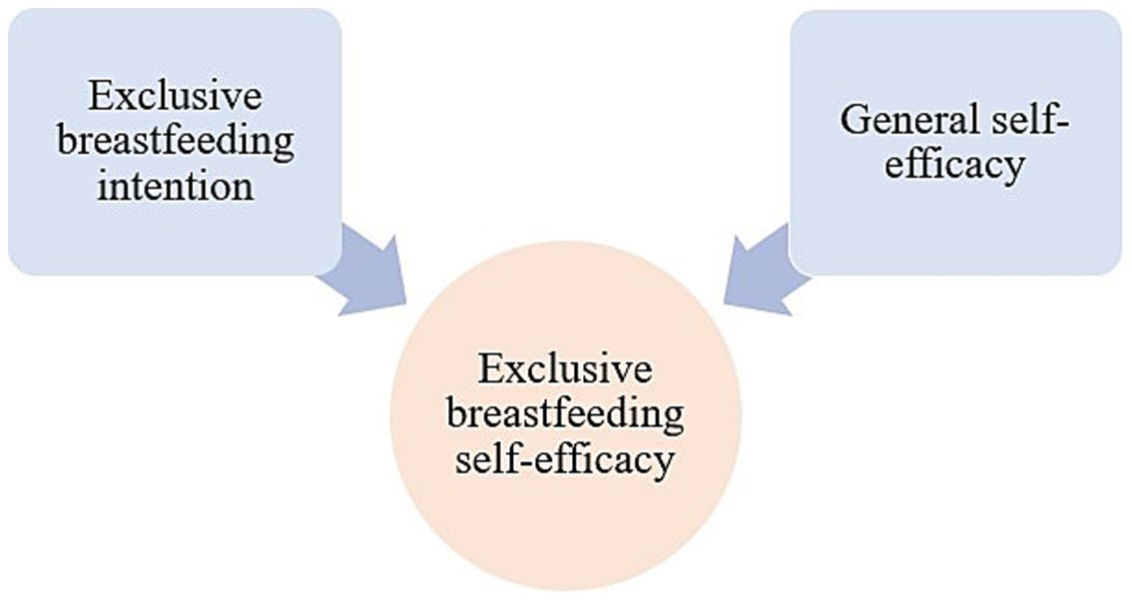
Conceptual model of prenatal exclusive breastfeeding self-efficacy predictors in AA women.

About 1.9, 20.8, and 77.4% of participants had low, medium, and high EBF self-efficacy scores, respectively. The mean EBF self-efficacy score of participants was 35.16 (SD = 7.41; range 9–45) and the mean general self-efficacy score was 33.56 (SD = 4.67; range 22–40). All independent variables were categorical variables except general self-efficacy. EBF self-efficacy, the dependent variable was a continuous variable. Data were assessed to ensure that the assumptions of one-way ANOVA (51) and bivariate correlation (52) test were met thereafter, these tests were performed in Statistical Package for the Social Sciences (v.28). Exclusive breastfeeding self-efficacy was significantly associated with EBF intention (*p* = 0.034) (Figure 2), and general self-efficacy (*r* = 0.387, *p* = *p* ≤ 0.001) (Table 3), but not associated with parity (Figure 3).

**Table 3:** Predictors of exclusive breastfeeding self-efficacy (*n* = 53).

Characteristic	*p*-value
Age	0.374
Marital status	0.377
Parity	0.470
Education	0.912
Employment	0.600
Exclusive breastfeeding intention	0.034*
General self-efficacy	0.001*

*Significant at *p* ≤ 0.05.

## 4. Discussion

The present study examined the validity and reliability of the BSES-EBF tool, and the relationship between exclusive breastfeeding self-efficacy and demographic variables. Findings revealed that the BSES-EBF instrument is valid and reliable to measure EBF self-efficacy in AA women. The positive association between EBF self-efficacy and general self-efficacy suggests that the BSES-EBF tool has construct validity. In addition, intention to breastfeed exclusively was positively associated with EBF self-efficacy in AA women, also suggesting that the BSES-EBF has criterion-related validity. At 1 month, Boateng et al. (28) reported Cronbach’s alpha coefficients of 0.82 and 0.85, and 0.77 and 0.79 at 3 months for the Cognitive and Functional sub-scales of the BSES-EBF, respectively. Similarly, the adapted BSES-EBF tool also had a Cronbach’s alpha coefficient of 0.86 among women in Egypt (29). In the present study, Cronbach’s alpha coefficient of 0.907 was reported, suggesting that BSES-EBF is a reliable tool to measure EBF self-efficacy, as a Cronbach’s alpha coefficient of 0.7 and above is generally considered acceptable (49, 50).

AA women had high prenatal EBF self-efficacy and general self-efficacy with means of 35.15 and 33.56, respectively. Similar finding was reported in previous studies that examined prenatal breastfeeding self-efficacy in AA women in the United States (12, 25). Conversely, in McCarter-Spaulding and Gore’s (27) study, AA women had the lowest postpartum breastfeeding self-efficacy scores compared with other women who identified as Black (African, Cape Verdean, Caribbean) (27). Similarly, compared with non-Hispanic White women, AA women had lower general self-efficacy scores (53). Assari (53) argued that the lower level of education and income of AA population compared with non-Hispanic White population explained the difference in general self-efficacy scores (53). In the present study, many participants (71.7%) had at least some college education, which may explain the different findings reported in this study compared with Assari’s study. Self-efficacy predicts self-esteem and persistence (54, 55); therefore, we may infer that AA women have a high self-esteem which is reflected in their strong determination to breastfeed exclusively. Indeed, in two studies – Ahmed and Rojjanasrirat (56) and Aderibigbe and Lucas (9), women who breastfed exclusively were reported to have strong determination and high breastfeeding self-efficacy (9, 56). The high EBF self-efficacy of AA women may reflect interventions to reduce breastfeeding disparities (57). Most participants (71.8%) had at least some college education, suggesting that more AA women are acquiring college education, similar to non-Hispanic White women, and that women with college education are more likely to participate in research compared with those with lower level of education (58). The level of education of AA women may also explain their high EBF self-efficacy as reported in a previous study (59). Finally, more than half of participants (50.9%) were relatively young, being within the age range of 18–30. The high EBF self-efficacy and general self-efficacy scores may also be attributed to the women’s age as younger women were reported to have higher self-efficacy compared to older women (60). The low sample size in the present study should be considered when interpreting inferences from this study.

Most women (81.1%) in this study planned to breastfeed exclusively. Conversely, McKinley and colleagues observed a significantly lower breastfeeding intention in AA women compared with non-Hispanic White women (26). As expected, EBF self-efficacy was significantly associated with general self-efficacy and exclusive breastfeeding intention, however, it was not associated with age, marital status, parity, education, and employment. Conversely, Ahmed et al. reported that EBF self-efficacy was significantly associated with age, education, and employment (29). Further exploration of the association between EBF self-efficacy and intention to breastfeed exclusively revealed that women who were undecided about infant feeding method and those who planned to feed their infants with formula only in the first weeks after birth had the lowest EBF self-efficacy scores.

### 4.1. Limitations

Preliminary literature review showed that no study has examined EBF self-efficacy and its predictors in AA women. Data collection over 8 months recruited 53 participants thus the sample size limits generalization. Previous studies reported that online surveys have low response rate compared with telephone or paper-based surveys (61). In addition, we received a comment posted on the Facebook ad warning women not to participate in our study and making reference to the Tuskegee study. Hence, the low sample size supports the assertion that AA persons may be wary of participating in research due to mistrust (62). Data were collected via an online survey which may have introduced a self-selection bias (63). However, to increase credibility, inclusion criteria were included in the survey to ensure that only participants who met the criteria had access to the survey. The cross-sectional design of the study may not have provided a robust assessment of the validity (especially predictive validity) and reliability of the BSES-EBF, compared to a longitudinal design as in Boateng et al. (28) where data were collected at 1 and 3 months postpartum. Further, the present study assessed all BSES-EBF items, providing no information about the Cognitive and Functional sub-scales of the tool.

### 4.2. Implications

Findings from this study have implications for research and clinical practice. We examined EBF self-efficacy nonetheless more information is required about the validity of the BSES- EBF scale to predict EBF (predictive validity). Therefore, future longitudinal studies should assess the relationship between EBF self-efficacy and EBF practice after giving birth to their infants in AA women and in other population with low EBF rates using a larger sample size. Additionally, researchers should strive to maintain transparency and earn the trust of participants, especially AA population to facilitate increased research participation. Most items in the BSES- EBF and general self-efficacy scale focused on women’s ability to overcome difficulties. Previous studies reported that AA population have higher physical and psychological resilience compared with non-Hispanic White population (64, 65). Hoffman et al. (66) also found that half of White medical students and residents believed that “black people’s skin is thicker than White people’s skin” (p. 4296). Thus, they reported lower pain ratings for a black person compared to a White person (66) therefore, caution should be exercised when applying findings from this study such that interpretations of the high EBF-self efficacy and general self-efficacy of AA women do not suggest that AA women are monolithic, particularly because of the low sample size for this study. Lastly, intention to breastfeed exclusively was one of the predictors of EBF self-efficacy. Prenatal breastfeeding education increased breastfeeding self-efficacy postpartum among women (23). Hence, nurses and midwives should continue to emphasize the importance of feeding infants with only breast milk (education) for the first 6 months during antenatal classes. It is expected that this intervention might encourage more women to decide to breastfeed their infants exclusively for 6 months while leveraging on the current formula shortage in the United States.

## 5. Conclusion

The exclusive breastfeeding self-efficacy scale used in this study is valid and reliable to measure EBF self-efficacy in AA women. AA women had high exclusive breastfeeding self-efficacy, predicted by intention to breastfeed exclusively and general self-efficacy. Women who did not intend to breastfeed had the lowest EBF self-efficacy scores. Hence, the BSES-EBF tool is indeed valid to identify women with low confidence to breastfeed their infants exclusively after birth. Finally, only one component was extracted from the factor analysis, suggesting that there is only one latent variable (confidence to practice exclusive breastfeeding) underlying the BSES-EBF tool.

## Data availability statement

The raw data supporting the conclusions of this article will be made available by the authors, without undue reservation.

## Ethics statement

The studies involving humans were approved by University of Connecticut Institutional Review Board. The studies were conducted in accordance with the local legislation and institutional requirements. The participants provided their written informed consent to participate in this study.

## Author contributions

TA: conceptualization, methodology, literature review, and data collection. TA and SW: preliminary and final data analysis. TA: writing – original draft preparation. SW, WH, and RL: supervision, and writing – review and editing. All authors contributed to the article and approved the submitted version.

## Conflict of interest

The authors declare that the research was conducted in the absence of any commercial or financial relationships that could be construed as a potential conflict of interest.

## Publisher’s note

All claims expressed in this article are solely those of the authors and do not necessarily represent those of their affiliated organizations, or those of the publisher, the editors and the reviewers. Any product that may be evaluated in this article, or claim that may be made by its manufacturer, is not guaranteed or endorsed by the publisher.

## Abbreviations

AA: African American
BSES-EBF: breastfeeding self-efficacy scale to measure exclusive breastfeeding
EBF: Exclusive breastfeeding
SD: standard deviation
